# A Machine Learning Approach for Tracing Tumor Original Sites With Gene Expression Profiles

**DOI:** 10.3389/fbioe.2020.607126

**Published:** 2020-11-24

**Authors:** Xin Liang, Wen Zhu, Bo Liao, Bo Wang, Jialiang Yang, Xiaofei Mo, Ruixi Li

**Affiliations:** ^1^Key Laboratory of Computational Science and Application of Hainan Province, Haikou, China; ^2^Key Laboratory of Data Science and Intelligence Education, Ministry of Education, Hainan Normal University, Haikou, China; ^3^School of Mathematics and Statistics, Hainan Normal University, Haikou, China; ^4^Qingdao Geneis Institute of Big Data Mining and Precision Medicine, Qingdao, China; ^5^Geneis (Beijing) Co., Ltd., Beijing, China

**Keywords:** the ability of tissue tracing, random forest, naive Bayes, machine learning, uncertain origins

## Abstract

Some carcinomas show that one or more metastatic sites appear with unknown origins. The identification of primary or metastatic tumor tissues is crucial for physicians to develop precise treatment plans for patients. With unknown primary origin sites, it is challenging to design specific plans for patients. Usually, those patients receive broad-spectrum chemotherapy, while still having poor prognosis though. Machine learning has been widely used and already achieved significant advantages in clinical practices. In this study, we classify and predict a large number of tumor samples with uncertain origins by applying the random forest and Naive Bayesian algorithms. We use the precision, recall, and other measurements to evaluate the performance of our approach. The results have showed that the prediction accuracy of this method was 90.4 for 7,713 samples. The accuracy was 80% for 20 metastatic tumors samples. In addition, the 10-fold cross-validation is used to evaluate the accuracy of classification, which reaches 91%.

## Introduction

Tumors can develop in any part of body, and some tumors even can metastasize to other parts of the body from their primary sites after developing at a certain point. In general, the occurrence of tumors at primary sites and their metastatic sites could be found deferentially, and the primary origins of metastatic cancers can be identified within a short amount of time by clinical assessments (Chen and Chen, 2001). Histological and imaging techniques are mostly employed to identify the origin of metastatic tumors. However, in some cancer patients, physicians cannot find the primary origin of tumors even after comprehensive examinations and assessment studies of patients with standard methods. These tumors are called carcinomas with unknown primary (CUP). According to statistical data, there are approximately 150,000 new cases of CUPs annually in the United States and Europe, and the numbers are still increasing though. Currently, approximately one third of cancer patients would develop metastasis after initial diagnosis and/or post-operation treatment. In many of those patients, it is relatively difficult for physicians to identify the primary origins of the metastatic cancers (Oien, 2009; Pavlidis and Pentheroudakis, 2012). To our knowledge, 2–4% of CUPs (Susman et al., 2012) account for all metastatic cancer. Even through autopsy, the primary origin of CUPs is uncertain (Myung et al., 2001; Petrushev et al., 2011). Because of limited treatment plan for CUP patients, the treatment efficacy is often unpredictable, and those patients usually have poor prognosis (Sun and Zhang, 2006; Gupta et al., 2007; Carmeliet and Jain, 2011; Petrakis et al., 2013). The immunohistochemistry assay is usually considered to be a diagnostic method for CUP patients. However, it is time-consuming and subjective. Moreover, the diagnostic accuracy is around by 30% for CUP patients, which is not reliable to design a personalized treatment plan for CUP patients. Currently, most CUP patients received radiological therapy (Stoyianni et al., 2011) or broad-spectrum chemotherapy. However, these treatments are not effective and with intolerable complications, and the prognosis is relatively poor as well. Therefore, it is urgent to develop effective clinical intervention for CUP patients (Guntinas-Lichius et al., 2006; Pavlidis and Fizazi, 2009; Hainsworth and Greco, 2014). Nowadays, identifying the primary origin of malignant tumors is critical for designing a treatment plan in clinical practices.

The targeted therapy (Tsao et al., 2005; Hudis, 2007; Miller et al., 2007; Varadhachary et al., 2008; Anderson and Weiss, 2010; Boscolo-Rizzo et al., 2015) can be used for tumors after accurately identifying the primary origin, which could greatly improve the survivals. It has been proven in the Minnie Pearl Cancer Research Network Study (Pavlidis and Pentheroudakis, 2010; Molina et al., 2012). Immunohistochemically, the marker has also been an important instrument for identifying the primary origin of cancerous tissues (Monzon et al., 2009; MacReady, 2010; Massard et al., 2011; Hashimoto et al., 2012; Oien and Dennis, 2012; Kim et al., 2013; Tang et al., 2018). Furthermore, a diagnostic method has been proposed to predict the primary origin of malignant cancers by comparing the gene expression profiles from the primary origin and the metastasis tissue (Hoadley et al., 2014). Many researchers have systematically compared the characteristics of gene expression profile across different cancers (Joyce and Pollard, 2009). Therefore, it is feasible to compare the differential gene expression to predict the primary origin of malignant cancer. There are two commercial products approved by FDA, which are Tissue of Origin (TOO) and CancerTYPE ID. Both of them are developed on the basis of differential gene expressions to predict primary origins.

TOO is a product of array-based gene expression profiles. TOO can identify 2,000 genes and 15 types of tumors, including thyroid cancer, breast cancer, non-small cell lung cancer, pancreatic cancer, gastric cancer, colorectal cancer, liver cancer, bladder cancer, kidney cancer, non-Hodgkin’s lymphoma, melanoma, ovarian cancer, sarcoma, testicular germ cell tumor, and prostate cancer. The advantage of this product is that it prevents the subjective bias. It can objectively identify the primary origin of cancers no matter which is well-differentiated or not. However, TOO is time-consuming, which is not feasible for clinical practices (Brugarolas, 2007; Economopoulou et al., 2015).

CancerTYPE ID is a product that uses cancer samples based on RT-PCR data. In the study (Marquard et al., 2015), 578 labeled samples covering 39 tumor types were included in datasets for tracing origins. The results showed that there was no significant difference in the accuracy of predictions of cancer with primary or metastatic tumors. Secondly, RT-PCR was used to evaluate the 92-gene (Ma et al., 2005) expression of cancer cells from patients and then compared with labeled 50 tumors from databases to predict the primary origin of metastatic tumors and their subtypes (Pappa et al., 2006). CancerTYPE ID has been able to compare gene expression profiles from tumor samples to reference database with more than 2,000 labeled tumors, therefore identifying the most accurate results. However, CancerTYPE ID does not have the relatively good accuracy for pancreatic cancer, colorectal cancer, and gastroesophageal cancer.

Though the above two products have good performance for some types of cancers, two products are costly with up to $3000–$4000 (Pillai et al., 2011; Oien and Dennis, 2012; Economopoulou et al., 2015), and the accuracy is limited to other types of cancer as well. In order to facilitate the low-cost and high-efficiency product, our study aimed to use RNA-seq data, which are extracted from TCGA database, combining with random forest and naïve Bayes algorithms to develop a computational model.

## Results

**FIGURE 1:**
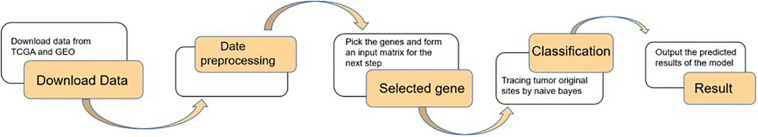
Flow chart of the article.

Firstly, data were downloaded from TCGA and GEO. Secondly, after data preprocessing for raw data, genes were selected by the random forest algorithm with 10-fold cross-validation. Finally, the naive Bayes classifier was used to classify the 20 kinds of tumors, and the output of the model was shown as the evaluation index. The detailed step is shown in Figure 1.

### Data Preparation

A total of 7,715 RNA-seq samples that covered 21 cancers and excluded metastatic cancers were extracted from TCGA. In the process of data preparation, we eliminated two samples due to the lack of clinical data. Therefore, the remaining 7,713 samples were used as either the training dataset or the validation dataset for the classification. Furthermore, the expression spectrum matrix of 7,633 samples was constructed. Each sample contained 20,501 genes. In this paper, 372 samples from metastatic cancers were selected as the test dataset, of which 352 samples belong to Skin Cutaneous Melanoma (SKCM). The ratio of SKCM was much higher than other types of metastatic cancers, and we excluded SKCM data from our selected data in order to reduce the possible effects on the results. The detailed information of selected data is shown in Table 1.

**TABLE 1:** Detailed information of data covering 21 cancers downloaded from TGCA.

**Cancer**	**Total samples**	**Samples from women**	**Samples from men**	**Percentage (%)**	**Note**
BLCA	301	80	221	3.9	
BRCA	1,056	1,044	11	13.7	1 person has no clinical information
CESC	258	258	0	3.3	
COAD	451	215	236	5.8	
GBM	153	53	100	2.0	
HNSC	480	128	352	6.2	
KIRC	526	184	342	6.8	
KIRP	222	63	159	2.9	
LAML	173	80	93	2.2	
LGG	439	192	247	5.7	
LIHC	294	99	195	3.8	
LUAD	486	262	224	6.3	
LUSC	428	109	319	5.5	
OV	261	261	0	3.4	
PAAD	142	64	78	1.8	
PRAD	379	0	379	4.9	
READ	153	70	82	2.0	1 person has no clinical information
SKCM	80	34	46	1.0	
STAD	415	147	268	5.4	
THCA	500	367	133	6.5	
UCEC	516	516	0	6.7	
Total	7,713	4,226	3,485	99.8	
					

For the independent validation dataset, 48 samples are obtained from GEO and processed according to the description in section “Materials and Methods” and then used for the trained naive Bayesian model to make the prediction. The detailed information of selected data is shown in Table 2.

**TABLE 2:** Detailed information of data covering five cancers downloaded from GEO.

**Cancer**	**Total samples**	**Percentage (%)**
LIHC	9	18.75
UCEC	6	12.5
THCA	8	16.67
BLCA	11	22.92
PAAD	14	29.17
Total	48	99.98

### Gene Selection by Random Forests

**FIGURE 2:**
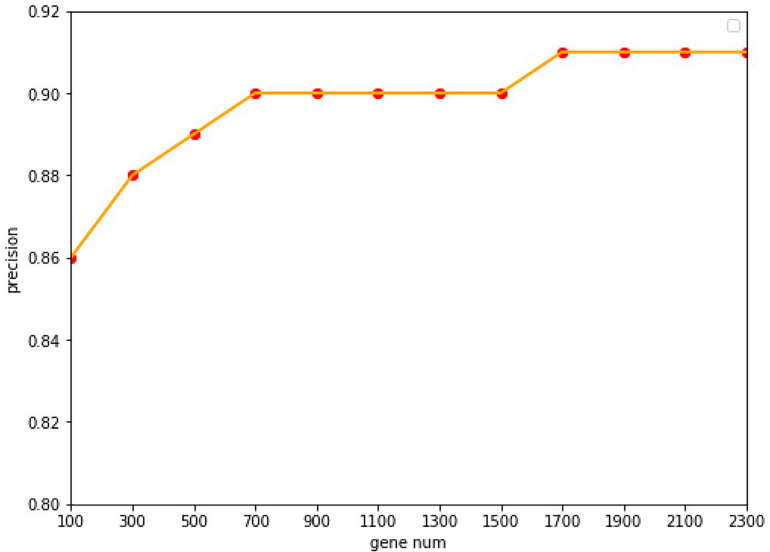
The accuracy with the different of number genes. With a 10-fold cross-validation accuracy, the value of the accuracy is increasing up to 1,700 genes, after which it keeps stable with the value of 91.07%.

Under the common condition, we use relatively low-cost panels but also include sufficient genes to determine the level of specific gene expression. However, the coverage of gene numbers would be significantly affected by the cost of panel. In order to reduce the cost of panels as well as improve the accuracy of tracing ability. Random forest algorithm was employed widely in the bioinformatics researches (Lv et al., 2019, 2020; Ru et al., 2019). In this study, the random forest algorithm was applied to select the features of the primary origin tumor samples, and a matrix of M^∗^N was formed, with M representing the number of samples and N representing the numbers of genes, and all samples were labeled with the type of each cancer. The expression profile was divided into 20 types of cancer, and the combination of five genes could be used to classify this problem (Ashburner et al., 2000). The Gini average impurity method of random forest was used as the standard to evaluate the importance of genes. The importance score of genes was obtained, and the genes were sequenced according to the score. We conducted many experiments, and the precision was the highest when 2,300 genes were obtained. The experimental results are shown in Figure 2. Our method takes five steps and increases N up to 2,300.

Based on the above analysis, genes with high scores were selected as the features, and 2,300 genes were extracted from each sample. Because some genes were not in the GEO database, we deleted these genes and got 2,284 genes. A 7,633^∗^2,284 matrix was constructed as the input matrix for cancer classification.

### Classification Based on Naive Bayes

**FIGURE 3:**
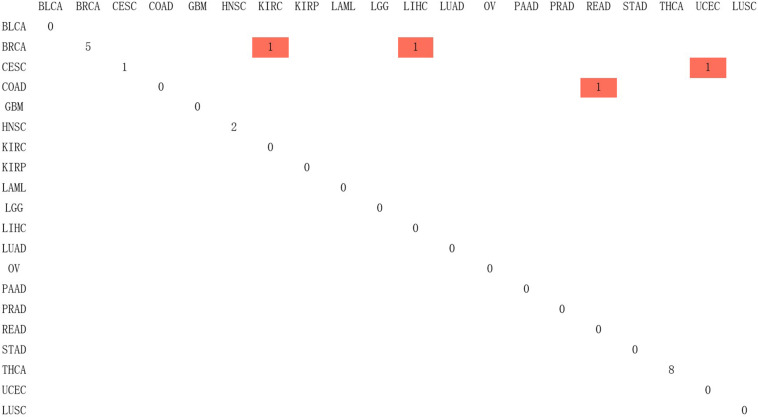
The confusion matrix of 2,284 genes in the classifier, in which red represented the result of inconsistency between primary and predicted cancer types.

Since Naive Bayes is relatively consistent for classification, this study used Naive Bayes as a classifier for genomic combination. In this study, we chose 75% of the dataset for training, and the remaining 25% was chosen for validation by using our model. The algorithm used gene expression as the feature for training and predicting the labeled cancer. After the training, the model achieved the accuracy of 91% in predicting the origin of the cancer. In order to validate the accuracy of classification of model for metastatic tumors, 20 metastatic tumors with known primary origin sites were applied to the model. 7,633^∗^2,284 was used as the input matrix for classification and applied to the naive Bayesian classification model to obtain the specific prediction results of specific cancer types with a prediction accuracy of 80% for metastatic cancer types, as shown in Figure 3.

**FIGURE 4:**
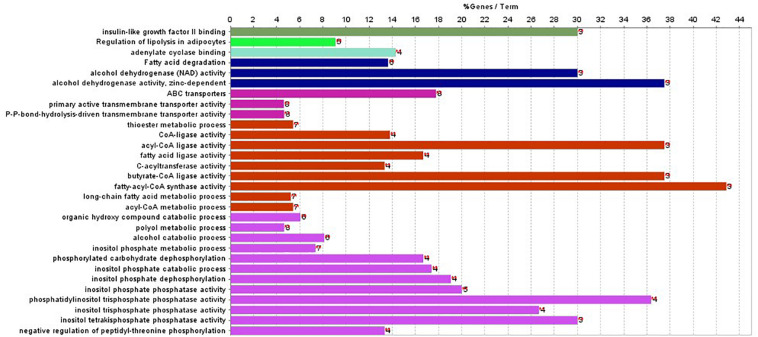
Gene enriched in biological process, cellular component, and molecular function were drawn for first 100-gene set by ClueGO.

In addition, ClueGO was used to identify gene ontology and enrichment analysis for selected genes. Due to the large number of 2,284 genes, we selected the top 100 genes with the highest score for analysis. The statistical significance level is set as the *p*-value of 0.001. The results of enrichment analysis are shown in Figure 4.

The enrichment results in Figure 4 show that the genes are significantly enriched in cellular metabolism, especially lipid metabolism. In addition, some genes are enriched in acetyl-CoA cycle, alcohol dehydrogenase NAD activity, etc. Almost all genes are enriched in lipid metabolism, which provides cellular energy for all cellular activity. Moreover, genes are also enriched through peroxisome proliferator-activated receptor (PPAR) signaling pathway. PPARs are nuclear hormone receptors activated by fatty acids and their derivatives and belong to ligand-activated receptors in the nuclear hormone receptor family. The PPAR signaling pathway plays a role in clearance of circulating lipid and promotes lipid oxidation and cell proliferation. The PPAR transcriptional activity can be regulated by non-gene crosstalks with phosphatases and kinases, including ERK1/2, p38-MAPK, PKC, and AMPK. The upregulated PPAR signaling pathway would lead to dysfunctional metabolic homeostasis and inflammatory response, ROS accumulation, as well as carcinogenesis across almost every tumor.

In order to further differentiate those 100 genes, the following heat map was drawn to further reveal the gene expression level in each cancer type.

**FIGURE 5:**
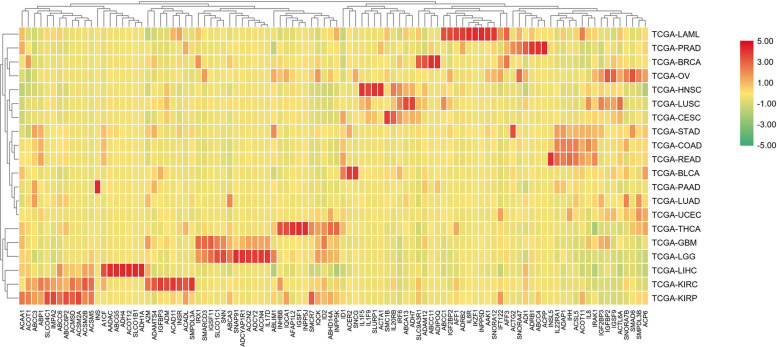
A heat map of the first 100 genes was screened by the random forest algorithm. Where, row is cancer type, column is gene. In this part, RPKM is used to define the gene expression level, and the average value of samples in each cancer type is calculated as the gene expression difference.

The analysis shown in Figure 5 reveals that there are expression differences of the first 100 genes in different cancers. Each small block represents a gene, and the color represents the size of gene expression. The higher level of the expression is represented with the darker color (red indicates upregulated and green indicates downregulated). The bottom horizontal line represents a different gene, while the vertical line on the right represents a different cancer.

### Independent Verification

**FIGURE 6:**
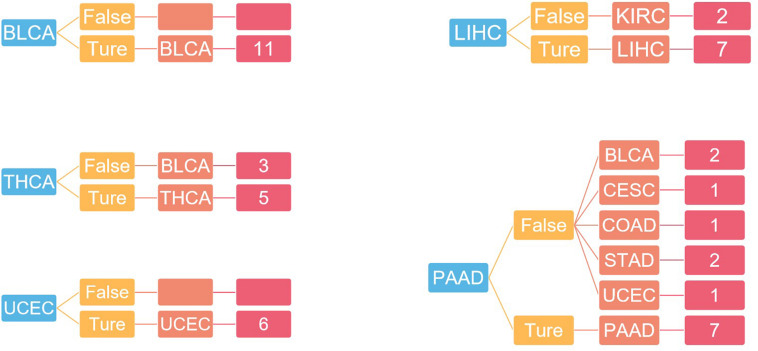
The result of independent verification. Blue represents the primary tumor, orange represents the accuracy of the prediction, light red represents the predicted tumor type, and dark red represents the number of predicted tumor types.

For independent tests, the model with the previous training parameters was tested on the dataset in GEO, and the probability of each sample being accurately assigned to each category was given, with an overall accuracy of 75%. The specific results are shown in Figure 6.

### Performance Assessment

**FIGURE 7:**
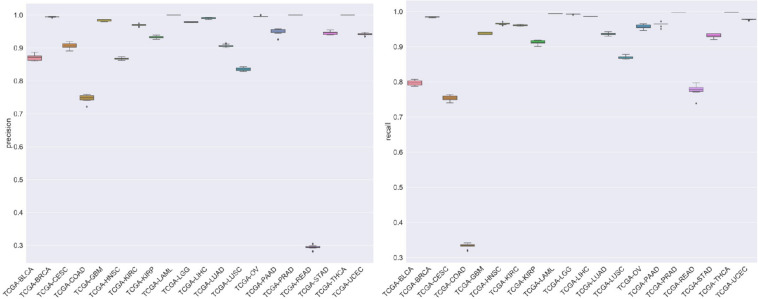
The figure represented the recalls and precision after 10-fold cross-validation.

For the evaluation of classification performance, this study used the 10-fold cross-validation for the algorithm with the feature in each gene set. To be specific, the samples were randomly divided into 10 subsets; 1 of 10 subsets was selected as the test set at one time, and the other 9 was merging to 1 training set. The accuracy of cross-validation is 90%, which indicated that the algorithm achieved a good performance. The precision, recalls, and f1 scores were used to evaluate the significance of the model as well. The detailed results are shown in Figure 7.

**FIGURE 8:**
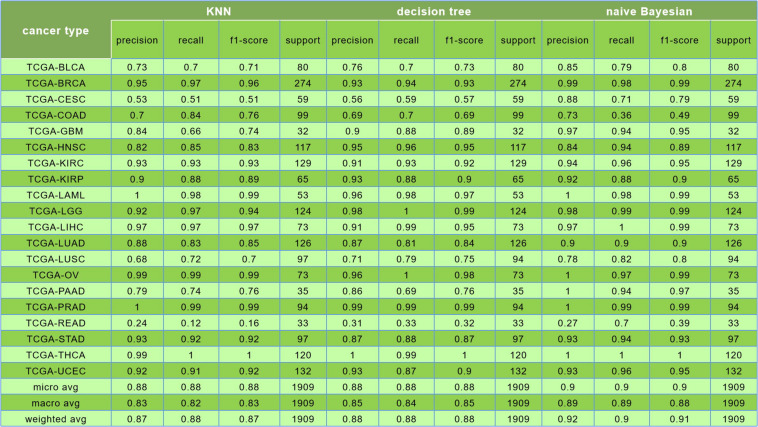
In this figure, the first was the result of k-nearest neighbor (*k* = 5) algorithm, and its prediction accuracy was only 88%; the second was the result of decision tree algorithm, and the classification accuracy was only 88%; the third is the result of naive Bayesian algorithm, and the classification accuracy was reaching to 90%.

The comparison among results of the *k*-nearest neighbor (*k* = 5), decision tree, and Naive Bayesian to classify 20 cancers is shown in Figure 8.

## Materials and Methods

### Data Preparation

The TCGA RNA-seq and array data were downloaded from the ICGC Data Portal^1^. Each sample and each gene from each cancer type table were extracted to generate a matrix of M^∗^N, where M is the number of samples, N is the number of genes, and all the samples were labeled by cancer types. All primary tumors were divided into training sets and metastatic tumors were divided into test sets.

For the independent set, 48 samples from 5 known cancer origin sites were downloaded from Gene Expression Omnibus (GEO). These tumors belong to GSE10907, GSE11222, GSE5608, GSE8352, GSE4895, GSE8912, GSE7966, and GSE12281. In addition, these 5 cancers belong to the 20 cancer types in this paper.

### Gene Selection

In order to reduce the cost of gene number determined by gene panel, in this study, random forest algorithm was applied to select genes. The Gini average impurity in random forest was used as the criterion to estimate the importance of genes. The random forest is composed of several decision trees, which are binary decision trees. Each node in the decision tree is a condition on a single gene. As a result, we can achieve the goal by splitting the dataset into two datasets; therefore, a similar expression level can be classified in the same dataset. For random forest, the average reduction of each feature impurity can be calculated. In addition, the importance score of genes can be calculated and sorted according to the score. GI stands for Gini, S stands for importance score, G = {g_1_,g_2_,.,g_n_} stands for feature, and C stands for cancer type. That is, to calculate the Gini score S_j_ for each feature g_j_, the calculation formula of Gini index is as follows:

$${\text{GI}}_{\text{m}}=1-\sum_{\text{c}=1}^{\left|\text{C}\right|}{\text{P}}_{\text{mk}}^{2}$$

where c represents C categories, and P_mk_ represents the proportion of category k in node m.

The importance of feature g_j_ in node m, that is, the variation of Gini impurities before and after node m branch, is calculated as follows:

$${\text{S}}_{\text{jm}}={\text{GI}}_{\text{m}}-{\text{GI}}_{\text{l}}-{\text{GI}}_{\text{r}}$$

where GI_l_ and GI_r_, respectively, represent the Gini index of the two new nodes after branching, and S_jm_ represents the importance of feature g_j_ in node m.

If the node m with characteristic g_j_ that appears in decision tree i belongs to M, the importance of g_j_ in the ith tree is calculated as follows:

$${\text{S}}_{\text{ij}}=\sum_{\text{m}\in \text{M}}{\text{S}}_{\text{jm}}$$

Assuming the random forest has t trees, the importance score formula of forest is:

Sj*=∑i=1t∑m∈MSjm

The importance score is obtained by normalizing all the importance scores obtained:

Sj=Sj*∑i=1nSi

The top N genes with high scores were selected until the stopping criterion was met. Finally, the selected genes in all samples participated in the next classification.

### Enrichment

Using the gene ontology (Bindea et al., 2009; Gene Ontology Consortium, 2019) as the database of enrichment analysis and annotating the function of specific gene sets to analyze their biological significance, ClueGO (Zhao et al., 2014) is used for visualization.

### Classification

In this paper, naive Bayes was used as the classifier of gene combination. Naive Bayes is one of the classical machine learning algorithms. It is a classification algorithm based on Bayes theorem. Its principle is simple and easy to implement. The core idea of naive Bayesian algorithm is to assume that each feature is independent. For a given type of data to be judged, classify and predict according to the training dataset, and calculate the probability that the current type of data to be judged belongs to a certain category through Bayesian theorem. The maximum probability relationship obtained is that the algorithm judges the category of these data. Naive Bayesian algorithm can be divided into three parts:

First, determine the feature attributes; that is to say, the expression profiles of 2,284 genes corresponding to each sample were extracted. Then, it was assumed that all the features conformed to the Gauss distribution. The samples in the dataset were labeled as cancer type. G represents the characteristics and C represents the type of cancer, which can be calculated as the prior probability P(C). C_k_ represents the kth category, g_i_ represents the ith feature, and then calculate conditional probability by prior probability. The formula is as follows:

$$\text{P}\left(\text{G}|{\text{C}}_{\text{k}}\right)=\text{P}\left({\text{G}}_{1}={\text{g}}_{1},{\text{G}}_{2}={\text{g}}_{2},\cdots ,{\text{G}}_{\text{n}}={\text{g}}_{\text{n}}|{\text{C}}_{\text{k}}\right)$$

The conditional probability of all the kth classes is calculated by the Bayesian formula:

$$\text{P}\,\left({\text{C}}_{\text{k}}|\text{G}\right)=\frac{\text{P}\,\left(\text{G}|{\text{C}}_{\text{k}}\right)\,\text{P}\,\left({\text{C}}_{\text{k}}\right)}{\text{P}\,\left(\text{G}\right)}=\text{P}\,\left({\text{C}}_{\text{k}}\right)\,\prod_{\text{i}=1}^{\text{n}}\text{P}\,\left({\text{g}}_{\text{i}}|{\text{C}}_{\text{k}}\right)$$

Since all the features conform to the Gaussian distribution and are independent of each other, the formula for conditional probability becomes as follows:

$$\text{P}\,\left(\text{G}|{\text{C}}_{\text{k}}\right)=\prod_{\text{i}=1}^{\text{n}}\text{P}\,\left({\text{g}}_{\text{i}}|{\text{C}}_{\text{k}}\right)=\prod_{\text{i}=1}^{\text{n}}\text{P}\,\left({\text{g}}_{\text{i}}|\mu_{\text{i},{\text{C}}_{\text{k}}},\sigma_{\text{i},{\text{C}}_{\text{k}}}\right)$$

$$=\frac{1}{\sigma_{\text{i},{\text{C}}_{\text{k}}}\,\sqrt{2\,\pi }}\,\exp\left\{-\frac{{\left({\text{g}}_{\text{i}}-\mu_{\text{i},{\text{C}}_{\text{k}}}\right)}^{2}}{2\,\sigma_{\text{i},{\text{C}}_{\text{k}}}^{2}}\right\}$$

where g_i_ is the ith feature, and μ_i,ck_ and σ_i,ck_ are the mean and variance of the ith feature in the K class C_k_, respectively.

The conditional probability formulas for all the Kth class are calculated as follows:

$$\text{P}\,\left({\text{C}}_{\text{k}}|\text{G}\right)=\frac{\text{P}\,\left(\text{G}|{\text{C}}_{\text{k}}\right)\,\text{P}\,\left({\text{C}}_{\text{k}}\right)}{\sum_{\text{k}}\text{P}\,\left(\text{G}|{\text{C}}_{\text{k}}\right)\,\text{P}\,\left({\text{C}}_{\text{k}}\right)}\propto \text{P}\,\left(\text{G}|{\text{C}}_{\text{k}}\right)\,\text{P}\,\left({\text{C}}_{\text{k}}\right)$$

Finally, obtain the relationship between the maximum probability data to be classified and the category, P(C_k_| G), that is:

$$\text{y}={\text{argmax}}_{{\text{C}}_{\text{k}}}\,\text{P}\,\left(\text{G}|{\text{C}}_{\text{k}}\right)\,\text{P}\,\left({\text{C}}_{\text{k}}\right)$$

It is meaningful to indicate that we could get the most probable type of cancer under certain gene expressions.

## Discussion

In Figure 3, 20 known primary tumors were predicted, while 4 of them were misjudged, which may be related to the naive Bayesian algorithm. Naive Bayes is one of the few algorithms based on probability theory, which is a very simple and convenient algorithm. However, the premise of this algorithm is to assume that each feature is independent of others, which is not in line with the reality. Therefore, it may produce errors in the classification results, leading to the decline of the prediction accuracy. In addition, in Figure 3, COAD was mislabeled as READ. It was possibly because the anatomical proximity is relatively close and may share differential gene expression. During the normal digestive process, the function of colon and rectum is not significantly different, while colon may contribute to maintaining the gut microenvironment. The epithelial cells that are usually changed in colon adenocarcinoma and rectum adenocarcinoma are not well-distinguished. It may possibly increase both the subjective and objective bias of our model. One case of CESC was misdiagnosed as UCES. Those two female malignant tumors are more commonly regulated by the female hormone, which share similar risk factors. The anatomical proximity is close as well. The above cases indicated that anatomical proximity may share oncogenic genes to drive genetic mutation, such as both cancers contain KRAS mutations (Gene Ontology Consortium, 2019), or it is difficult to differentiate epithelial or adrenal cell changes before oncogenesis. It is critical to point out that some biological factors might bring some effect for model performance. It is necessary to be considered as the model construction. In addition, there are only 20 cases of known primary tumor data used to predict the classification. The data size is relatively small, so we cannot get a certain conclusion. We need to further expand the database for classification and prediction.

In Figure 4, the first 100 genes with the highest score are selected by the random forest algorithm. Some genes obtained by this method may have high correlation; that is to say, these genes will provide the same information for the classifier. In addition, although we used the 10-fold cross-validation to investigate the performance of the model, in the independent validation, the accuracy of this model is only 75%. The predictive error to PAAD is large, and the independent validation dataset is small.

## Conclusion

In this study, the random forest and naive Bayesian algorithms were employed to trace the origin of CUP sites. Through a large number of experiments, we found that 2,284 genes with the highest score achieved the best performance. Performance evaluation shows that this method can achieve good classification and prediction results. In addition, ClueGO enrichment analysis was used for the top 100 genes with the highest scores. The results showed that some genes were enriched in PPAR signaling pathway. Upregulation of PPAR signaling pathway has been proven to lead to metabolic homeostasis disorder, inflammation, ROS accumulation, and carcinogenesis. In summary, the proposed approach can reduce the cost and has high efficiency, and thus it is promising for clinical practices.

## Data Availability Statement

Publicly available datasets were analyzed in this study. This data can be found here: https://dcc.icgc.org/releases/release_26/.

## Author Contributions

JY, BL, and WZ conceived the project. XL and BW implemented the experiments and analyzed the data. XL, XM, and RL prepared the data and performed literature search. XL and JY wrote the manuscript. All authors approved the final manuscript.

## Conflict of Interest

BW, JY, and XM were employed by company Geneis (Beijing) Co., Ltd. The remaining authors declare that the research was conducted in the absence of any commercial or financial relationships that could be construed as a potential conflict of interest.

## Footnotes

1
https://dcc.icgc.org/releases/release_26/

## References

[B1] Anderson G. G. Weiss L. M. (2010). Determining tissue of origin for metastatic cancers: meta-analysis and literature review of immunohistochemistry performance. *Appl. Immunohistochem. Mol. Morphol.* 18 3–8. 10.1097/pai.0b013e3181a75e6d 19550296

[B2] Ashburner M. Ball C. A. Blake J. A. Botstein D. Butler H. Cherry J. M. (2000). Gene ontology: tool for the unification of biology. The gene ontology consortium. *Nat. Genet.* 25 25–29. 10.1038/75556 10802651 PMC3037419

[B3] Bindea G. Mlecnik B. Hackl H. Charoentong P. Tosolini M. Kirilovsky A. (2009). ClueGO: a Cytoscape plug-in to decipher functionally grouped gene ontology and pathway annotation networks. *Bioinformatics* 25 1091–1093. 10.1093/bioinformatics/btp101 19237447 PMC2666812

[B4] Boscolo-Rizzo P. Schroeder L. Romeo S. Pawlita M. (2015). The prevalence of human papillomavirus in squamous cell carcinoma of unknown primary site metastatic to neck lymph nodes: a systematic review. *Clin. Exp. Metast.* 32 835–845. 10.1007/s10585-015-9744-z 26358913

[B5] Brugarolas J. (2007). Renal-cellcarcinoma: molecularpathways and therapies. *N. Engl. J. Med.* 356 185–187. 17215538 10.1056/NEJMe068263

[B6] Carmeliet P. Jain R. K. (2011). Principles and mechanisms of vessel normalization for cancer and other angiogenic diseases. *Nat. Rev. Drug Discov.* 10 417–427. 10.1038/nrd3455 21629292

[B7] Chen L. M. Chen B.-S. (2001). A robust adaptive DFE receiver for DS-CDMA systems under multipath fading channels. *IEEE Trans. Signal Process.* 49 1523–1532. 10.1109/78.928705

[B8] Economopoulou P. Mountzios G. Pavlidis N. Pentheroudakis G. (2015). Cancer of unknown primary origin in the genomic era: elucidating the dark box of cancer. *Cancer Treat. Rev.* 41 598–604. 10.1016/j.ctrv.2015.05.010 26033502

[B9] Gene Ontology Consortium (2019). The Gene ontology resource: 20 years and still going strong. *Nucleic Acids Res.* 47 D330–D338. 10.1093/nar/gky1055 30395331 PMC6323945

[B10] Guntinas-Lichius O. Peter Klussmann J. Dinsh S. Dinh M. Schmidt M. Semrau R. (2006). Diagnostic work-up and outcome of cervical metastases from an unknown primary. *Acta Otolaryngol.* 126 536–544. 10.1080/00016480500417304 16698706

[B11] Gupta G. P. Perk J. Acharyya S. de Candia P. Mittal V. Todorova-Manova K. (2007). ID genes mediate tumor reinitiation during breast cancer lung metastasis. *Proc. Natl. Acad. Sci. U.S.A.* 104 19506–19511. 10.1073/pnas.0709185104 18048329 PMC2148319

[B12] Hainsworth J. D. Greco F. A. (2014). Gene expression profiling in patients with carcinoma of unknown primary site: from translational research to standard of care. *Virchows Arch.* 464 393–402. 10.1007/s00428-014-1545-2 24487792

[B13] Hashimoto K. Sasajima Y. Ando M. Yonemori K. Hirakawa A. Furuta K. (2012). Immunohistochemical profile for unknown primary adenocarcinoma. *PLoS One* 7:e31181. 10.1371/journal.pone.0031181 22299055 PMC3267772

[B14] Hoadley K. A. Yau C. Wolf D. M. Cherniack A. D. Tamborero D. Ng S. (2014). Multiplatform analysis of 12 cancer types reveals molecular classificationwithin and across tissues of origin. *Cell* 158 929–944. 10.1016/j.cell.2014.06.049 25109877 PMC4152462

[B15] Hudis C. A. (2007). Trastuzumab: mechanism of action and use in clinical practice. *N. Engl. J. Med.* 357 39–51. 10.1056/nejmra043186 17611206

[B16] Joyce J. A. Pollard J. W. (2009). Microenvironmental regulation of metastasis. *Nat. Rev. Cancer* 9 239–252. 10.1038/nrc2618 19279573 PMC3251309

[B17] Kim K. W. Krajewski K. M. Jagannathan J. P. Nishino M. Shinagare A. B. Hornick J. L. (2013). Cancer of unknown primary sites: what radiologists need to know and what oncologists want to know. *AJR Am. J. Roentgenol.* 200 484–492. 10.2214/ajr.12.9363 23436835 PMC3603700

[B18] Lv Z. Jin S. Ding H. Zou Q. (2019). A random forest sub-Golgi protein classifier optimized via dipeptide and amino acid composition features. *Front. Bioeng. Biotechnol.* 7:215. 10.3389/fbioe.2019.00215 31552241 PMC6737778

[B19] Lv Z. Zhang J. Ding H. Zou Q. (2020). RF-PseU: a random forest predictor for RNA Pseudouridine sites. *Front. Bioeng. Biotechnol.* 8:134. 10.3389/fbioe.2020.00134 32175316 PMC7054385

[B20] Ma X. J. Patel R. Wang X. Salunga R. Murage J. Desai R. (2005). Molecular classification of human cancers using a 92-gene real-time quantitative polymerase chain reaction assay. *Arch. Pathol. Lab. Med.* 130 465–473. 10.5858/2006-130-465-MCOHCU 16594740

[B21] MacReady N. (2010). NICE issues guidance on cancer of unknown primary. *Lancet Oncol.* 11:824 10.1016/s1470-2045(10)70215-1 20842777

[B22] Marquard A. M. Birkbak N. J. Thomas C. E. Favero F. Krzystanek M. Lefebvre C. (2015). Tumor tracer: a method to identify the tissue of origin from the somatic mutations of a tumor specimen. *BMC Med. Genom.* 8:58. 10.1186/s12920-015-0130-0 26429708 PMC4590711

[B23] Massard C. Loriot Y. Fizazi K. (2011). Carcinomas of an unknown primary origin–diagnosis and treatment. *Nat. Rev. Clin. Oncol.* 8 701–710. 10.1038/nrclinonc.2011.158 22048624

[B24] Miller K. Wang M. Gralow J. Dickler M. Cobleigh M. Perez E. A. (2007). Paclitaxel plus bevacizumab versus paclitaxel alone for metastatic breast cancer. *N. Engl. J. Med.* 357 2666–2676. 10.1056/nejmoa072113 18160686

[B25] Molina R. Bosch X. Auge J. M. Filella X. Escudero J. M. Molina V. (2012). Utility of serum tumor markers as an aid in the differential diagnosis of patients with clinical suspicion of cancer and in patients with cancer of unknown primary site. *Tumour Biol.* 33 463–474. 10.1007/s13277-011-0275-1 22161237

[B26] Monzon F. A. Lyons-Weiler M. Buturovic L. J. Rigl C. T. Henner W. D. Sciulli C. (2009). Multicenter validation of a 1500-gene expression profile for identification of tumor tissue of origin. *J. Clin. Oncol.* 27 2503–2508. 10.1200/jco.2008.17.9762 19332734

[B27] Myung J. Kim K. B. Lindsten K. Dantuma N. P. Crews C. M. (2001). Lak of proteasome active site allostery as revealed by subunit-specific inhibitors. *Mol. Cell* 7 411–420. 10.1016/s1097-2765(01)00188-5 11239469

[B28] Oien K. A. (2009). Pathologic evolution of unknown primary cancer. *Semin. Oncol.* 36 8–37. 10.1053/j.seminoncol.2008.10.009 19179185

[B29] Oien K. A. Dennis J. L. (2012). Diagnostic work-up of carcinoma of unknown primary: from IHC to molecular profiling. *Ann. Oncol.* 23(Suppl. 10), x271–x277. 22987975 10.1093/annonc/mds357

[B30] Pappa K. I. Choleza M. Markaki S. Giannikaki E. Kyroudi A. Vlachos G. (2006). Consistent absence of BRAF mutations in cervical and endometrial cancer despite KRAS mutation status. *J. Gynecol. Oncol.* 100 596–600. 10.1016/j.ygyno.2005.09.029 16256179

[B31] Pavlidis N. Fizazi K. (2009). Carcinoma of unknown primary(CUP). *Crit. Rev. Oncol. Hematol.* 69 271–278. 10.1016/j.critrevonc.2008.09.005 18977667

[B32] Pavlidis N. Pentheroudakis G. (2010). Cancer of unknown primary site: 20 questions to be answered. *Ann. Oncol.* 21(Suppl. 7), vii303–vii307. 20943633 10.1093/annonc/mdq278

[B33] Pavlidis N. Pentheroudakis G. (2012). Cancer of unknown primary site. *Lancet* 379 1428–1435. 22414598 10.1016/S0140-6736(11)61178-1

[B34] Petrakis D. Pentheroudakis G. Voulgaris E. Pavlidis N. (2013). Prognostication in cancer of unknown primary (CUP): development of a prognostic algorithm in 311 cases and review of the literature. *Cancer Treat. Rev.* 39 701–708. 10.1016/j.ctrv.2013.03.001 23566573

[B35] Petrushev B. Tomuleasa C. Susman S. Soris̨ãu O. Aldea M. Kacsó G. (2011). The aixs of evil in the fight against cancer. *Rom. J. Intern. Med.* 49 319–325. 22568277

[B36] Pillai R. Deeter R. Rigl C. T. Nystrom J. S. Miller M. H. Buturovic L. (2011). Validation and reproducibility of a microarray-based gene expression test for tumor identification in formalin-fixed, paraffin-embedded specimens. *J. Mol. Diagn.* 13 48–56. 10.1016/j.jmoldx.2010.11.001 21227394 PMC3070545

[B37] Ru X. Li L. Zou Q. (2019). Incorporating distance-based top-n-gram and random forest to identify electron transport proteins. *J. Proteom. Res.* 18 2931–2939. 10.1021/acs.jproteome.9b00250 31136183

[B38] Stoyianni A. Pentheroudakis G. Pavlidis N. (2011). Neuroendocrine carcinoma of unknown primary: a systematic review of the literature and a comparative study with other neuroendocrine tumors. *Cancer Treat. Rev.* 37 358–365. 10.1016/j.ctrv.2011.03.002 21481536

[B39] Sun X. F. Zhang H. (2006). Clinicopathological significance of stromal variables: angiogenesis, lymphangiogenesis, inflammatory infiltration, MMP and PINCH in colorectal carcinomas. *Mol. Cancer* 5:43. 10.1186/1476-4598-5-43 PMC1618857 17026740

[B40] Susman S. Tomuleasa C. Soritau O. Mihu C. Rus-Ciuca D. Sabourin J. C. (2012). The colorectal cancer stem-like cell hypothesis: a pathologist’s point of view. *J. BUON* 17 230–236. 22740198

[B41] Tang W. Wan S. Yang Z. Teschendorff A. E. Zou Q. (2018). Tumor origin detection with tissue-specific miRNA and DNA methylation markers. *Bioinformatics* 34 398–406. 10.1093/bioinformatics/btx622 29028927

[B42] Tsao M. S. Sakurada A. Cutz J. C. Zhu C. Q. Kamel-Reid S. Squire J. (2005). Erlotinib in lung cancer: molecular and clinical predictors of outcome. *N. Engl. J. Med.* 353 133–144. 16014883 10.1056/NEJMoa050736

[B43] Varadhachary G. R. Raber M. N. Matamoros A. Abbruzzese J. L. (2008). Carcinoma of unknown primary with a colon-cancer profile-changing paradigm and emerging definitions. *Lancet Oncol.* 9 596–599. 10.1016/s1470-2045(08)70151-7 18510991

[B44] Zhao X. Zou Q. Liu B. Liu X. (2014). Exploratory predicting protein folding model with random forest and hybrid features. *Curr. Proteom.* 11 289–299. 10.2174/157016461104150121115154

